# Supplementary material for: Daptomycin Pharmacokinetics in Blood and Wound Fluid in Critical Ill Patients with Left Ventricle Assist Devices
Source: Antibiotics (Basel). 2023 May 13;12(5):904. doi: 10.3390/antibiotics12050904 (PMC10215934; doi:10.3390/antibiotics12050904)
Supplement: Supplementary file 1 [file antibiotics-12-00904-s001.zip › antibiotics-2352730-supplementary.pdf]

Supplementary Data

Material and Methods

Calibrator Samples, Quality Control Samples and Internal Standard.

All used solvents were of HPLC or comparable quality, and all reagents were of an analytical grade. Daptomycin was purchased from MSD Sharp & Dohme GmbH (Cubicin®) as a regular vial containing powder for reconstitution. Human serum was purchased from Sigma-Aldrich (Steinheim, Germany). Ultrapure water was obtained from Fresenius Kabi (Ampuwa®, Frankfurt Rhein-Main, Germany). Linezolide was obtained from Fresenius Kabi as a regular solution containing 2 mg/mL. Daptomycin was prepared by dissolving 350 mg of powder for injection in 1000 mL of ultrapure water to obtain a concentration of 350 mg/L. The internal standard was dissolved in an acetonitrile/methanol (1:1) mixture at a concentration of 20 mg/L.

Serum calibration standards with 5.0, 10, 25, 50, and 100 mg/L and serum quality control concentrations of 25 mg/L (low concentration, LQC), 50 mg/L (medium concentration, MQC), and 100 mg/L (high concentration, HQC) were prepared by adding appropriate volumes of a daptomycin stock solution to human serum. All solutions were stored as 500 µL aliquots in polypropylene Eppendorf tubes at -80° C and were thawed just before use.

Sample Preparation

To determine daptomycin in the serum, protein precipitation was performed by adding 200 µL of an acetonitrile/methanol (1:1) mixture with 20 mg/L of linezolide in 100 µL of patient serum. Subsequently, samples were mixed for 10 s, then centrifuged at 8000 g for 3 min. Next, 100 µL of the resulting supernatant was further diluted with 500 µL of water with 0.1% formic acid.

HPLC Conditions

An aliquot of 100 µL was injected into the HPLC–UV (Shimadzu, Duisburg, Germany) system and equipped with a diode array detector (SPD-M20A). Chromatographic analysis was performed using a reverse phase column MultoHigh 100 RP8 with a 5µm particle size (125 mmx3mm CS-Chromatographie Service GmbH, Lengerwehe, Germany) in combination with a column guard (Phenomenex, Aschaffenburg, Germany). Separation was performed using a gradient of 0.1% formic acid, 10% water in acetonitrile, and 0.1% formic acid in water with a flow rate of 1.0 mL/min. Daptomycin was monitored at a wavelength of 370 nm with a retention time of 9.9 min.

Assay Validation

For peak identification, aqueous solutions of daptomycin (100 mg/L) were analyzed with the above-mentioned method and were assessed for their intensity (area), shape, and retention time. Further assay validation was performed with a spiked human serum. The linearity of total daptomycin was conducted via a calibration curve with serum calibration standards. Each of the serum calibration standards was analyzed six times and evaluated by the peak area vs. target concentration, with an acceptable correlation coefficient of >0.95. The LOQ was set at 2.5 mg/L; this also corresponded to a signal-to-noise ratio of 10:1. The LOD was estimated at a signal-to-noise ratio of 3:1. To determine assay precision, the coefficient of variation (CV, reported in %) in serum quality control concentrations was calculated. Differentiation between the inter- and intraday precision was performed in addition, with intraday precision (repeatability) and was determined three times during the same day, while interday precision (reproducibility) was determined once a day for eight consecutive days. A general coefficient of variation of <15% was accepted. Accuracy was evaluated using the same samples as described, for precision, at three different concentrations on eight different days. The degree of accuracy was determined by the bias, with 15% being acceptable. The recovery was determined at 100 mg/l and 10 mg/l in six replicates each. The absolute recoveries (%) were calculated by comparing the peak areas of daptomycin in spiked human serum, which were extracted as described, with those of spiked aqueous solutions at the same concentration levels not subjected to the extraction procedure.

Furthermore, interference with other drugs was assessed by the cross-examination of 40 chromatograms of patients who did not receive daptomycin or linezolide but other anti-infectives and other intensive care-specific drugs, as well as six chromatograms of different blank sera.

The chromatograms were examined for interfering peaks at a retention time and detection wavelength of 370 nm for daptomycin. Additionally, a complete UV spectrum was checked for peak purity and identification.

Results of the validation

Chromatograms

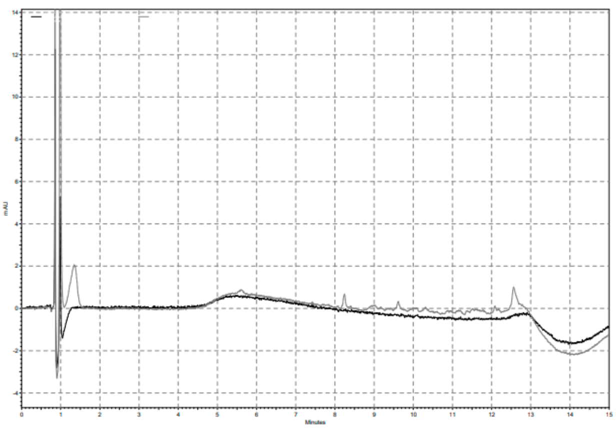

Serum blank sample (grey curve 300 nm, black curve 370 nm).

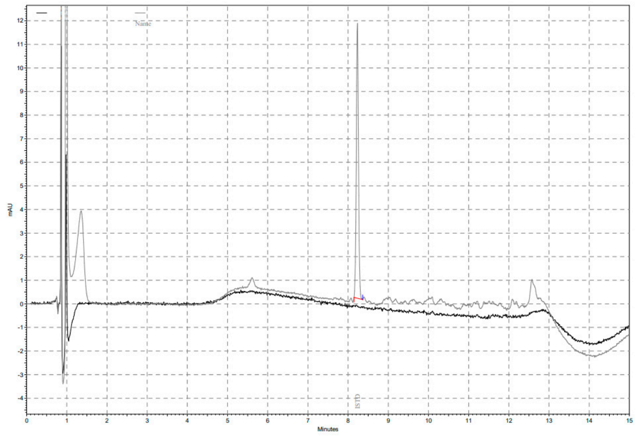

Serum blank sample with an internal standard (grey curve 300 nm, black curve 370 nm).

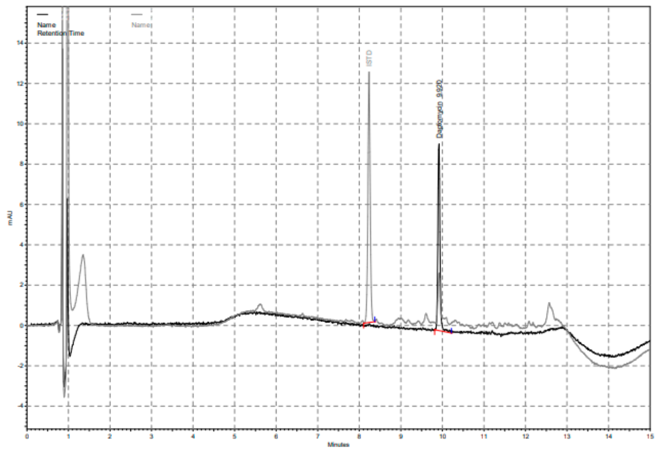

Serum calibration sample of 50 mg/l daptomycin (grey curve 300 nm, black curve 370 nm).

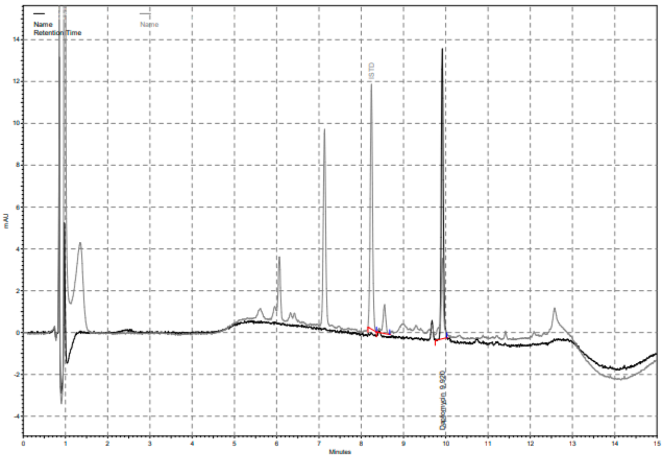

Optional: serum patient sample with daptomycin (grey curve 300 nm, black curve 370 nm).

Linearity

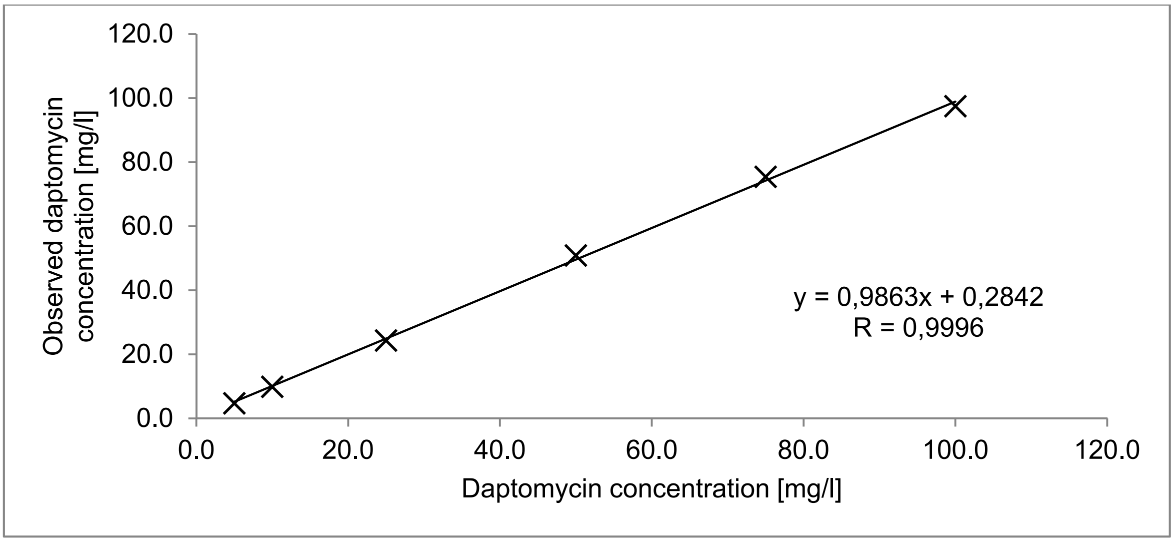

Calibration curve of daptomycin calibration standards ranging from 5 to 100 mg/l (R = 0,9996).

Precision and accuracy

Theoretical concentration [mg/L]	Mean concentration [mg/L]	Intraday [%]	Interday [%]	Bias [%]
100	98.5	3.7	5.0	- 1.6
50	50.6	3.7	4.4	+ 1.1
25	25.4	7.1	5.3	+ 1.6

Results of intraday and interday precision (CV%) and accuracy (bias%) of daptomycin in human serum.

The recovery rate for both concentrations was found to be around 100% (102% for the lower and 105% for the higher concentrations). LOQ was set at 2.5 mg/L and LOD at 1 mg/L.

Stability

Daptomycin has a reasonable overall stability, as described already by other authors (1)[xx]. For the validation, stability was tested in human serum and water for injection at different conditions, including room temperature, refrigeration at 4° C, and freezing at -20° C as well as -80° C. For stability testing, two samples were thawed out and subsequently analyzed. Stability was defined by the time at which the samples remained at a concentration of > 90% for their baseline concentrations. We did not observe any variation in the peak area over 24 hours after extraction when the autosampler was maintained at room temperature. Stock solutions were stable for at least 24 h at room temperature, for 5 days at 4°C, for 3 months at -20°C, and for 12 months at -80°C.Serum samples were stable for at least 24 h at room temperature, for 2 days at 4°C, for 3 months at -20°C, and 12 months at -80°C.

1. Ogami C, Tsuji Y, Kasai H, Hiraki Y, Yamamoto Y, Matsunaga K, Karube Y, To H. 2017. Evaluation of pharmacokinetics and the stability of daptomycin in serum at various temperatures. Int J Infect Dis 57:38-43.
